# Intelligence

**Published:** 1829-06

**Authors:** 


### INTELLIGENCE.

### MONTHLY REPORT OF PREVALENT DISEASES.

WE are not aware that any striking peculiarity has marked the diseases which have most prevailed since our last report. Scarlatina, measles, and hooping-cough still continue frequent, but are now less severe than they were during the month of April. We have seen one case of scarlatina, the progress and symptoms of which were somewhat unusual, and in which also the patient recovered under circumstances which seemed to justify an unfavorable prognosis.

A young lady, aged sixteen, was exposed to cold about ten days after the desquamation of the cuticle, subsequent to the eruption. She was at this time rapidly recovering her general health, which had been much subdued by the severe though short disease she had passed through. In a few hours after, she complained of having "caught cold," and of feeling an uncomfortable stiffness of the neck, headach, and slight feverish symptoms. The next morning, the whole of the anterior part of the throat was highly inflamed, particularly the integuments over the lower part of the left sterno-cleido-mastoideus muscle. The patient appeared dull and heavy, and towards evening she became nearly comatose. She was roused even by a loud noise but for a moment, and the only expression of complaint that could be drawn from her was that her hands and legs were very painful. They were slightly anasarcous, as was also the face. In this state she remained for three or four days, and it was concluded from the symptoms that effusion had taken place into the ventricles of the brain, and a guardedly unfavorable prognosis was given. A well-defined phlegmonous abscess had in the mean time formed nearly over the thyroid cartilage, which was opened on the fifth day from the first appearance of the external inflammation, and about four ounces of well-conditioned pus was discharged. From this time the symptoms of cerebral oppression gradually subsided, and the patient slowly recovered. The treatment adopted upon the first appearance of the comatose symptoms consisted of active purgatives, cold evaporating lotions constantly applied to the shaven scalp, a blister between the shoulders, and pills of squill, digitalis, and calomel. The general condition of the patient appeared to contraindicate the abstraction of blood.

In all cases of a similar kind which terminate favorably, it is impossible to determine positively that effusion had taken place in the ventricles of the brain. In the above instance the presumption is certainly strong in favor of such a supposition, both from the symptoms of cerebral oppression and the general anasarca which existed. We will not venture to assert that the cessation of the symptoms of cerebral affection depended upon the discharge of the matter from the abscess in the throat. The coincidence was, however, remarkable. Much diversity of opinion has existed as to the causes which especially favor the occurrence of anasarca after scarlatina. We believe it is universally admitted that it is usually the sequel of mild cases of the disease. In the case above related, there can be no doubt that exposure to cold was the exciting cause of the dropsical effusion, and, as far as our own observation goes, this is generally the case. The fact is undeniable that dropsy more frequently succeeds to scarlatina in cold than in hot or temperate climates, and in winter more often than at other seasons.

---

*Distribution of Prizes at the London University. Saturday, May 23d.*
The Marquis of Lansdowne in the chair.

An adjudication of prizes and certificates of honour took place in acknowledgment of talent and industry in the various branches of medical science. Every precaution had been adopted to secure the utmost impartiality in the distribution of the prizes to the young candidates. The honours and prizes were awarded by the result of answers in writing to prepared questions. A series of questions for the students of each professor was printed, of which a copy was delivered to the student after he came into the examination-room. The answers were written in the examination-room, and subsequently collected at one time. No book whatever was allowed to be brought into the room. The paper containing the answers was signed with a mark or motto, and the name of the student using it enclosed in a sealed envelope, inscribed with the mark or motto, was left with the warden, to be opened upon the day of the distribution of the prizes. In each class a gold medal and two silver medals were given, and certificates of honours to all who had attained a certain amount of excellence in their replies to the various questions. Upon the present occasion, the professors in succession read the motto affixed to the paper of answers which had been deemed the most meritorious, and to which consequently the gold medal had been awarded. The sealed packet was then opened by the warden, and the name of the successful competitor declared, who then presented himself to the noble chairman, by whom the certificate and prize were given. The professor next announced the mottos to which the silver medals had been awarded; and afterwards the names of the pupils were read who had obtained certificates of honours. The same student was allowed to be a competitor for a prize or certificate in every class.

Mr. George Atkinson, of Sheffield, obtained three gold medals, for physiology, medical practice, and midwifery; and the second silver medal for materia medica. To the honour of Mr. Atkinson, it should be stated that it was the first year of his attendance at any medical school.

Mr. Benjamin Phillips, of Newport, obtained two gold medals, for surgery and practical anatomy: and the first silver medal in another class, and the second silver medal for physiology.

Mr. Robert Gardner, of Staffordshire Potteries, received the gold medal for materia medica; the first silver medal for physiology; and the second silver medal for surgery.

Mr. John Jones, of Kidderminster, the gold medal for anatomy, and the second silver medal for practical anatomy.

Count Canares, eldest son of the Marquis Palmella, the gold medal for chemistry.

Mr. Frederick Duckham, of Falmouth, the first silver medal for materia medica; the first silver medal for practical anatomy; and the second silver medal for anatomy.

Mr. T. H. Connor, of London, the first silver medal for surgery.

Mr. W. M. Richards, of Norwood, Surrey, the first silver medal for the practice of medicine.

Mr. Alfred Warehouse, of Halifax, the first silver medal for midwifery.

Mr. E. J. Quekett, of Langport, the first silver medal for chemistry.

In addition to the above prizes, Professors Bennett and Thomson presented some books to those pupils who deserved some mark of public approbation and the esteem of their teachers, although they had not succeeded in obtaining the gold or silver medal.

We believe it is intended to publish the lists of questions which were submitted to the candidates by the various professors. We have seen the questions submitted by Professor Thomson on materia medica. The extent of information that must be possessed by those pupils who correctly answered the majority of them must have been very great.

The whole of the ceremony was highly gratifying, and no doubt can be entertained of the great advantages that will be obtained from the stimulus to exertion that will be secured on the part of the pupils by these public avowals of their merit. With the most sincere wishes for the future welfare and professional reputation of these youthful aspirants for honour and fame, we will conclude by urging them to remember the axiom of the talented Dr. Young, whose recent loss we have to deplore, that in every study "system is the Ariadnean thread, without which all is confusion."

---

*Lectures on Medical and General Botany, by* Mr. Gilbert Burnett, *Theatre of Anatomy, Great Windmill street.*

The general principles of botany, with the relations and purposes of the science, will be treated in the following order:

1. The natural philosophy of organization, with the comparative anatomy and physiology of vegetables.

2. The systematic arrangement or classification of plants, with reference more especially to the celebrated methods of Rayo, Linnæus, and Jussieu.

3. The products and properties of vegetables, both medical and economical. In this section the distinguishing characteristics of the most valuable and important plants will be pointed out; the officinal and poisonous species particularly described; and the descriptions illustrated by specimens and plates.

These lectures commenced on Tuesday, May 5th, at half-past six P.M. and will be continued every Thursday, Saturday, and Tuesday, at the same hour, until completed.

---

*Guy's Hospital. Vase presented to* BRANSBY COOPER, Esq. *by his Pupils, April 27th.*

The pupils of Guy's Hospital presented Mr. BRANSBY COOPER with a magnificent silver vase, which cost 95*l.* Mr. CHARLES GAZELEE was deputed by his fellow-students to express their sentiments on the occasion, which he did in the presence of the pupils and many members of the profession, assembled in the theatre to witness the ceremony. He addressed Mr. Cooper as follows:

"Sir: The gentlemen whose names are recorded on this scroll have deputed me to offer you their sincere congratulations on the success which attended your prosecution of the editor of the Lancet, for one of the most unjust and deliberate libels that ever issued from the press.

"We are, sir, well aware that, anxious as you must have been to wipe off the imputation from your character, you were not so much actuated by a feeling of personal consideration, as by a wish to support the respectability and dignity of the profession; and we look upon yours as a signal triumph of principle and justice over a most odious system of misrepresentation and detraction.

"So many are the difficulties with which the healing art is beset, and so constantly are the best and most experienced judgments liable to be deceived, that no profession affords so wide a scope for a designing mind to scatter its illiberal reflections, with a more specious pretext of plausibility; while none has a higher claim on that virtuous charity which would bid us extenuate rather than blazen forth the misfortunes of others. Nay, sir, where is the man who will dare to say that he never committed an error? 'Let him that is without fault cast the first stone.' But, when even the truth is misrepresented, what character, however eminent, is secure? To be traduced by tongues which, though they have not the candour to speak the truth, yet will be the chronicles of men's doings:

'Tis but the fate of place, and the rough brake
That virtue must go through. We must not stint
Our necessary actions, in the fear
To cope malicious censurers.'

"That the recreant defamer who has once quaffed the chalice of slander can never be again so well satiated as by revelling in the lifeblood of some murdered reputation, is not very surprising; but such a system calls loudly for correction. Under a mistaken and misnamed form of 'liberality,' it is calculated to rear up distrust instead of confidence, dissention instead of unanimity, discord instead of harmony, enmity instead of friendship; it would break the bonds of private endearment, and unknit every social tie.

"It is, sir, in acknowledgment of the generous spirit which impelled you to come forward at such a crisis, when the feelings of a whole profession had been outraged by a deeply practised slanderer, and in acknowledgment of the success which crowned your exertions, that we would now offer our tribute; and, as a more lasting pledge of our unalienated good opinion, and of the sincerity of our congratulations, I beg leave, in the names of my fellow-students, to request your acceptance of this vase, which bears an inscription expressive of the object for which it was presented:

To Bransby Blake Cooper, Esq.
The Pupils of Guy's Hospital
present this Vase,
To testify their ardent participation in his Triumph
over a daring and malicious Libel.
1829.

"I trust, sir, that you will overlook the imperfect manner in which the humble individual who has the honour to present it now discharges the office with which his fellow-pupils have intrusted him. I am sure that I need not appeal to your class for permission to thank you for the zeal which, in common with the other professors of this school, you evince in the discharge of the duties connected with it, and by which you maintain with credit the fame established by your distinguished predecessors."

Mr. Cooper replied nearly in the following terms:

"Gentlemen: I have often had occasion to express my sense of your approbation and kindness. At present I can find no language adequate to convey to you the feelings I experience upon this further testimony of your approbation, and of the very flattering terms of your address to me.

"Whatever pain and mortification I may have suffered from the attacks of malevolence and envy, I can assure you I have found an ample compensation for those sufferings in the opportunity they have given me of learning your sentiments, and the estimation in which I have the honour to be held by those who are most intimately acquainted with my professional acquirements and my private character.

"Gentlemen, you have done me no more than justice in ascribing the efforts I have made to vindicate myself from the attacks in question more to a sense of what was due to the honour of my profession, than to any personal feeling or interest of my own.

"I am vain enough to think that no person acquainted with me, or with the progress of my professional career, could have been influenced by the libels otherwise than by feelings of contempt for the author. With regard to those to whom I was unknown, I was well aware there was more risk than advantage in bringing my name before the public in connexion with the revolting detail of a surgical operation, liable to be misunderstood by the ignorant, and easily misrepresented by the malicious.

"But I felt, as I am glad to find you feel, that the honour and character of the medical profession itself were attacked, and its utility as a liberal profession diminished by the system of slander which had been too long allowed to pass unnoticed; and I resolved, at whatever hazard to myself, to seize the occasion which was given me of appealing to the laws of my country, and of exposing to the public the mischievous, base, and sordid means which were employed to depreciate and defame the profession to which I belong.

"Gentlemen, I beg you to accept my heartfelt thanks for your splendid gift, which is to me inestimable. I shall preserve and cherish it as a testimony of your kindness and good opinion, the possession of which must ever be an ornament in prosperity, a consolation in adversity. I shall make it my study to cultivate and deserve your good opinion; and I shall transmit this splendid vase to my children, that in aftertimes they may judge from this pledge how their father was estimated by his pupils."

Dr. C. J. ROBERTS has been elected one of the physicians to the General Dispensary, Aldersgate street; Dr. WOODFORD having resigned.

### LITERARY NOTICE.

Dr. KENNEDY has in forward preparation for the press a work which will form three volumes 8vo. entitled "A History of the Medical Sciences, Biographical and Philosophical; containing an Account of the Persons and Writings that have conduced to the Improvement of Physic, from its Origin in Britain to the end of the Eighteenth Century."

### METEOROLOGICAL JOURNAL,

By Messrs. HARRIS and Co. Mathematical Instrument Makers, 50, High Holborn.

| April | Rain gauge. | Moon. | Thermom. 9 A.M. | Thermom. MAX. | Thermom. MIN. | Barometer. 9 A.M. | Barometer. 10P.M. | De Luc's Hygrom. 9A.M. | De Luc's Hygrom. 10P.M. | Winds. 9 A.M. | Winds. 10P.M. | Atmospheric Variations. 9 a.m. | Atmospheric Variations. 2 p.m. | Atmospheric Variations. 10 p.m. |
|---|---|---|---|---|---|---|---|---|---|---|---|---|---|---|
| 20 | .16 | | 49 | 56 | 40 | 29.57 | 29.71 | 57 | 52 | WNW | WNW | Cloudy | Fine | Fine |
| 21 | | | 47 | 53 | 45 | .68 | .53 | 53 | 60 | WSW | E | Foggy | Fine | Cloudy |
| 22 | | | 46 | 55 | 44 | .29 | .39 | 62 | 61 | E | ENE | Rain | Rain | Show'ry |
| 23 | .34 | | 50 | 55 | 45 | .54 | .59 | 61 | 65 | ENE | ESE | Foggy | Fine | Fine |
| 24 | | | 49 | 50 | 40 | .59 | .69 | 67 | 68 | E | NE | Foggy | Rain | Cloudy |
| 25 | | | 40 | 50 | 38 | .62 | .79 | 70 | 73 | ENE | NE | Rain | Rain | Fine |
| 26 | .35 | ☾ | 42 | 51 | 35 | .73 | .64 | 65 | 62 | ENE | ENE | Fine | Fine | Fine |
| 27 | | | 43 | 51 | 43 | .61 | .60 | 62 | 57 | WSW | NNW | Show'ry | Fine | Show'ry |
| 28 | | | 47 | 51 | 39 | .57 | .28 | 57 | 55 | W | NW | Cloudy | Rain | Fine |
| 29 | .12 | | 46 | 50 | 35 | .61 | .67 | 50 | 52 | NW | N | Fine | Fine | Fine |
| 30 | | | 42 | 52 | 38 | .73 | .64 | 52 | 53 | NW | NNW | Fine | Fine | Show'ry |
| May | | | | | | | | | | | | | | |
| 1 | | | 47 | 57 | 48 | .57 | .60 | 55 | 53 | WNW | W | Cloudy | Fine | Fine |
| 2 | | | 53 | 60 | 46 | .60 | .63 | 50 | 52 | W | WSW | Fine | Fine | Fine |
| 3 | | ● | 53 | 60 | 47 | .67 | .69 | 53 | 55 | W | W | Fine | Cloudy | Cloudy |
| 4 | | | 56 | 60 | 47 | .72 | .90 | 55 | 50 | W | W | Fine | Fine | Fine |
| 5 | | | 59 | 61 | 54 | .95 | .92 | 50 | 53 | SW | WSW | Fine | Cloudy | Cloudy |
| 6 | | | 57 | 62 | 46 | .86 | .91 | 55 | 52 | SW | WSW | Rain | Fine | Fine |
| 7 | | | 53 | 63 | 46 | .90 | .97 | 52 | 53 | W | WNW | Show'ry | Rain | Fine |
| 8 | | | 51 | 62 | 50 | 30.07 | 30.06 | 53 | 50 | W | W | Fine | Fine | Fine |
| 9 | | | 57 | 63 | 52 | .06 | 29.98 | 50 | 50 | W | NW | — | — | — |
| 10 | | ☽ | 58 | 68 | 50 | 29.94 | .92 | 48 | 48 | SE | SE | — | — | — |
| 11 | | | 60 | 63 | 53 | .93 | .91 | 47 | 49 | ESE | E | — | — | — |
| 12 | | | 61 | 65 | 47 | .87 | .85 | 49 | 45 | E | E | — | — | — |
| 13 | | | 59 | 64 | 50 | .86 | .88 | 45 | 45 | E | ESE | — | — | — |
| 14 | | | 60 | 67 | 54 | .88 | .88 | 45 | 45 | S | WSW | — | — | — |
| 15 | | | 60 | 66 | 51 | .89 | .90 | 45 | 50 | WSW | ESE | — | — | — |
| 16 | | | 56 | 69 | 47 | .99 | .99 | 50 | 50 | E | E | Cloudy | Fine | Fine |
| 17 | | | 52 | 63 | 49 | .99 | .95 | 48 | 46 | ENE | ENE | Fine | — | — |
| 18 | | ○ | 55 | 62 | 46 | .95 | .89 | 45 | 46 | ESE | E | — | — | — |
| 19 | | | 54 | 66 | 48 | .83 | .80 | 46 | 44 | E | E | — | — | — |

The quantity of Rain fallen in the month of April, was 3 inches and 79-100ths.

